# Supplementary material for: Short-Term Associations of Ambient Fine Particulate Matter (PM2.5) with All-Cause Hospital Admissions and Total Charges in 12 Japanese Cities
Source: Int J Environ Res Public Health. 2021 Apr 13;18(8):4116. doi: 10.3390/ijerph18084116 (PMC8070111; doi:10.3390/ijerph18084116)
Supplement: Supplementary file 1 [file ijerph-18-04116-s001.pdf]

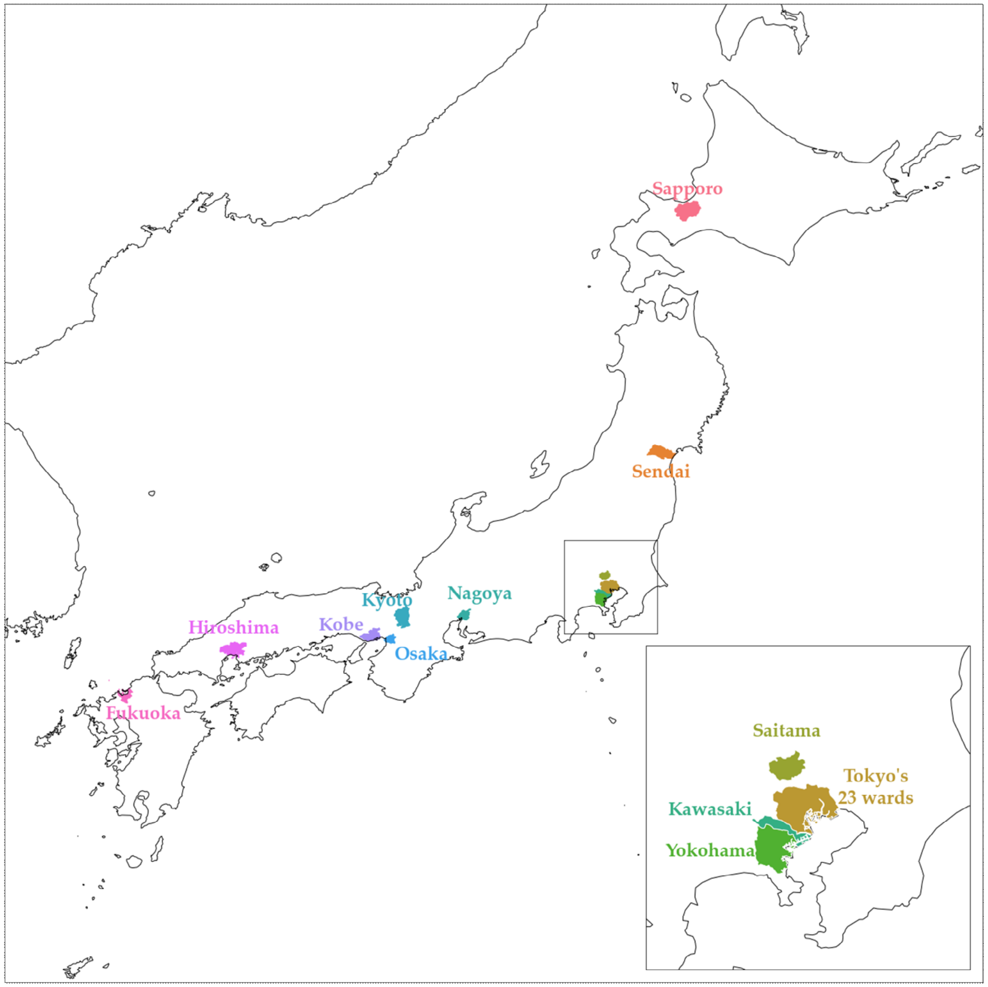

Suppl. Fig. S1. Map of the 12 Japanese cities included in this study

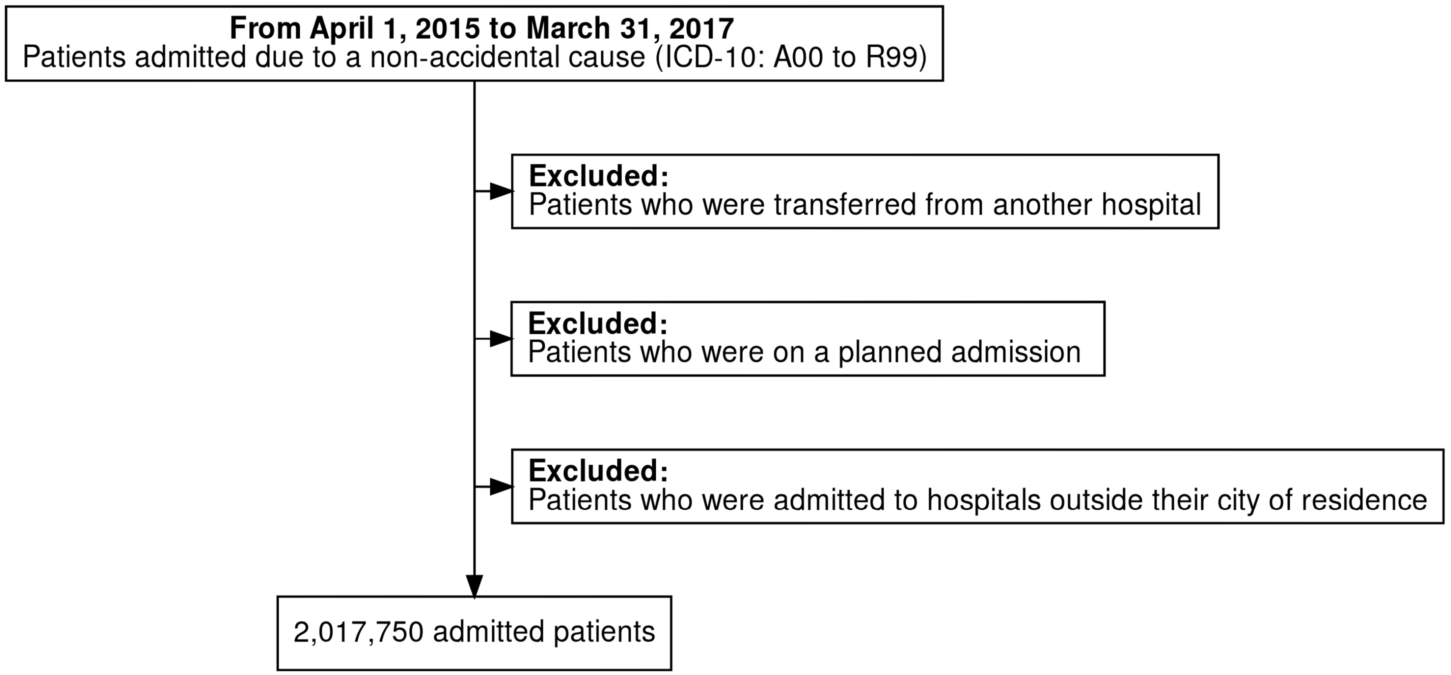

Suppl. Fig. S2. Patient selection flow chart.

Note: As we obtained only the last data for analysis, the exact number of patients in each step was unknown.

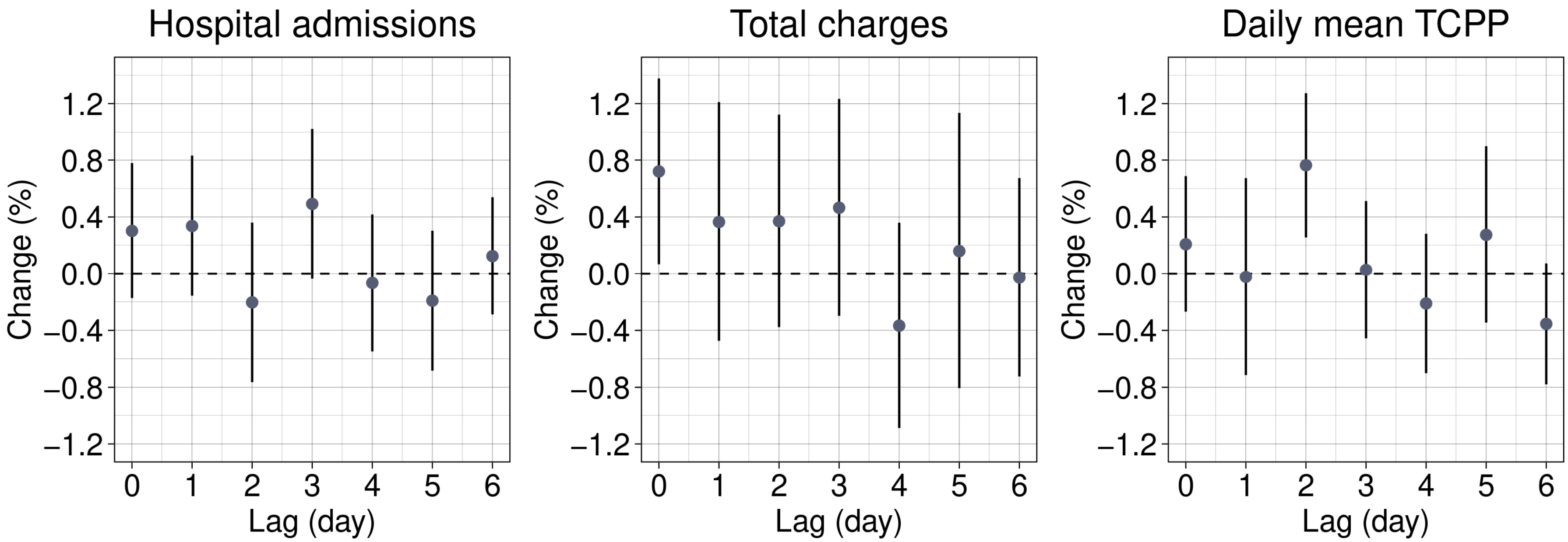

Suppl. Fig. S3. National average percent change of all-cause hospital admissions, total charges, and daily mean total charges per patient with a 10-µg/m^3 increase in the ambient $PM_{2.5}$ concentration estimated by the unconstrained distributed lag model. TCPP: total charges per patient.

Suppl. Table S1. City-specific cover ratios estimated by data from hospital bed function reports (HBFRs) in 2018

City	DPC hospitals[a]			Non-DPC hospitals			Beds			EC beds			Patients[b]			EC patients[c]		
	Total	Included	%	Total	Included	%	Total	Included	%	Total	Included	%	Total	Included	%	Total	Included	%
Sapporo	37	36	97.3	141	101	28.4	28,459	15,656	55.0	21,019	14,831	70.6	399,540	325,282	81.4	128,365	106,278	82.8
Sendai	17	17	100.0	30	23	23.3	9,513	7,748	81.4	8,647	7,514	86.9	192,572	178,266	92.6	72,240	67,029	92.8
Saitama	6	6	100.0	20	14	30.0	5,388	3,972	73.7	4,405	3,495	79.3	107,177	92,547	86.3	45,027	37,540	83.4
Tokyo's 23 wards	105	104	99.0	305	250	18.0	69,507	46,707	67.2	57,250	45,388	79.3	1,460,568	1,262,744	86.5	491,732	426,847	86.8
Yokohama	38	38	100.0	77	63	18.2	21,708	15,328	70.6	17,777	14,455	81.3	474,673	422,187	88.9	176,677	161,851	91.6
Kawasaki	14	14	100.0	20	16	20.0	8,500	6,243	73.4	7,262	6,161	84.8	181,288	160,943	88.8	58,616	53,105	90.6
Nagoya	24	24	100.0	95	77	18.9	18,669	12,330	66.0	14,580	11,729	80.4	363,980	320,144	88.0	130,840	119,813	91.6
Kyoto	21	21	100.0	74	58	21.6	17,588	10,826	61.6	13,265	10,387	78.3	267,572	232,465	86.9	94,037	81,107	86.3
Osaka	45	44	97.8	128	103	19.5	30,269	20,132	66.5	24,168	19,031	78.7	563,555	480,631	85.3	200,868	174,183	86.7
Kobe	25	25	100.0	70	47	32.9	14,023	9,432	67.3	11,234	8,721	77.6	255,525	223,039	87.3	90,259	81,790	90.6
Hiroshima	16	16	100.0	59	44	25.4	11,178	7,237	64.7	7,693	6,402	83.2	169,277	150,142	88.7	60,830	55,283	90.9
Fukuoka	26	26	100.0	75	49	34.7	17,316	12,716	73.4	12,696	11,068	87.2	295,332	262,111	88.8	93,306	80,600	86.4

[a]Hospitals that were under the DPC/PDPS. [b]Newly admitted patients from July 1, 2015 to June 30, 2016. [c]Newly admitted EC patients who were not transferred or planned admissions from July 1, 2015 to June 30, 2016. DPC: Diagnosis Procedure Combination, DPC/PDPS: Diagnostic Procedure Combination/Per-Diem Payment System, EC: emergency care.

Suppl. Table S2. City-specific percent change in all-cause hospital admissions with a 10-μg/m^3 increase in ambient $PM_{2.5}$ concentrations

City	Lag 0			Lag 1			Lag 2			Lag 3			Lag 01		
	PC[a]	95%CI	*p*	PC[a]	95%CI	*p*	PC[a]	95%CI	*p*	PC[a]	95%CI	*p*	PC[a]	95%CI	*p*
Sapporo	2.66	0.89–4.45	0.003	2.50	0.67–4.34	0.007	1.30	−0.45–3.08	0.146	1.95	0.29–3.62	0.021	3.53	1.43–5.67	<.001
Sendai	−0.47	−2.23–1.32	0.604	−0.50	−2.33–1.35	0.592	0.66	−1.12–2.48	0.468	0.93	−0.79–2.67	0.292	−0.57	−2.62–1.51	0.588
Saitama	1.28	−0.51–3.11	0.162	0.56	−1.27–2.42	0.550	−0.33	−2.15–1.51	0.723	−0.92	−2.68–0.86	0.308	1.36	−0.81–3.56	0.221
Tokyo's 23 wards	0.46	−0.31–1.23	0.242	0.18	−0.61–0.97	0.662	−0.35	−1.14–0.45	0.394	−0.08	−0.86–0.71	0.842	0.46	−0.47–1.39	0.333
Yokohama	0.87	−0.26–2.02	0.132	0.78	−0.38–1.95	0.187	−0.05	−1.20–1.13	0.940	0.82	−0.33–1.99	0.163	1.14	−0.21–2.51	0.098
Kawasaki	0.69	−0.69–2.07	0.328	0.09	−1.31–1.51	0.898	−1.07	−2.47–0.35	0.140	0.59	−0.81–2.01	0.407	0.58	−1.06–2.24	0.492
Nagoya	0.58	−0.56–1.73	0.322	0.21	−0.93–1.35	0.722	0.65	−0.47–1.78	0.256	−0.01	−1.11–1.11	0.993	0.54	−0.79–1.89	0.427
Kyoto	0.56	−0.74–1.88	0.396	0.76	−0.54–2.06	0.253	0.71	−0.56–1.99	0.275	0.51	−0.75–1.78	0.428	0.85	−0.62–2.35	0.259
Osaka	−0.21	−1.19–0.78	0.673	0.39	−0.59–1.38	0.439	0.42	−0.56–1.41	0.403	0.34	−0.64–1.33	0.496	0.11	−1.01–1.25	0.842
Kobe	−0.35	−1.71–1.03	0.617	0.43	−0.94–1.81	0.541	0.37	−0.97–1.73	0.591	0.17	−1.16–1.51	0.803	0.04	−1.52–1.62	0.961
Hiroshima	−0.11	−1.51–1.31	0.880	0.72	−0.72–2.17	0.329	0.56	−0.85–1.99	0.439	0.54	−0.85–1.95	0.446	0.38	−1.21–2.00	0.639
Fukuoka	−0.02	−1.23–1.21	0.981	−0.14	−1.36–1.09	0.823	−0.07	−1.27–1.13	0.903	−0.11	−1.28–1.07	0.852	−0.08	−1.48–1.35	0.916

[a]Adjusted for calendar time, temperature, relative humidity, public holidays, and the day of the week. PC: percent change.

Suppl. Table S3. City-specific percent change in the total charges with a 10-μg/m³ increase in ambient $PM_{2.5}$ concentrations

City	Lag 0			Lag 1			Lag 2			Lag 3			Lag 01		
	PC[a]	95%CI	*p*	PC[a]	95%CI	*p*	PC[a]	95%CI	*p*	PC[a]	95%CI	*p*	PC[a]	95%CI	*p*
Sapporo	2.85	0.23–5.54	0.033	4.56	1.82–7.37	0.001	2.98	0.37–5.66	0.025	2.76	0.30–5.29	0.028	5.05	1.90–8.30	0.002
Sendai	0.60	−2.20–3.48	0.677	−0.38	−3.26–2.58	0.798	2.62	−0.30–5.62	0.079	1.66	−1.09–4.49	0.238	0.10	−3.16–3.48	0.951
Saitama	0.52	−2.26–3.39	0.716	0.17	−2.66–3.09	0.906	0.14	−2.69–3.06	0.923	−0.59	−3.33–2.24	0.680	0.57	−2.77–4.03	0.740
Tokyo's 23 wards	0.05	−1.07–1.19	0.924	0.08	−1.06–1.24	0.887	−0.01	−1.16–1.16	0.988	0.21	−0.92–1.36	0.715	0.10	−1.26–1.47	0.888
Yokohama	1.72	−0.01–3.49	0.052	1.56	−0.20–3.35	0.083	1.42	−0.35–3.21	0.117	1.18	−0.55–2.94	0.181	2.25	0.19–4.35	0.033
Kawasaki	2.16	0.10–4.28	0.040	1.09	−1.00–3.22	0.310	0.15	−1.94–2.28	0.890	1.26	−0.81–3.37	0.234	2.34	−0.13–4.88	0.064
Nagoya	1.02	−0.66–2.72	0.235	1.04	−0.63–2.74	0.223	1.43	−0.23–3.12	0.091	0.22	−1.41–1.88	0.789	1.43	−0.54–3.43	0.155
Kyoto	1.27	−0.77–3.36	0.224	1.75	−0.31–3.85	0.096	1.12	−0.90–3.19	0.278	0.24	−1.75–2.27	0.814	1.96	−0.38–4.36	0.102
Osaka	0.88	−0.50–2.28	0.210	0.17	−1.21–1.56	0.814	0.40	−0.98–1.80	0.572	−0.01	−1.37–1.38	0.992	0.68	−0.90–2.28	0.402
Kobe	−0.47	−2.44–1.55	0.647	−0.76	−2.75–1.27	0.462	−0.43	−2.41–1.59	0.674	−1.15	−3.09–0.83	0.253	−0.84	−3.10–1.47	0.473
Hiroshima	−0.21	−2.44–2.06	0.852	0.74	−1.52–3.05	0.525	0.99	−1.25–3.29	0.388	0.29	−1.93–2.56	0.800	0.33	−2.20–2.92	0.802
Fukuoka	0.96	−0.89–2.85	0.309	1.83	−0.03–3.72	0.054	0.29	−1.51–2.12	0.753	−0.39	−2.16–1.42	0.671	1.91	−0.24–4.12	0.082

[a]Adjusted for calendar time, temperature, relative humidity, public holidays, and the day of the week. PC: percent change.

Suppl. Table S4. City-specific percent change in the daily mean of total charges per patient with a 10-μg/m^3 increase in ambient $PM_{2.5}$ concentrations

City	Lag 0			Lag 1			Lag 2			Lag 3			Lag 01		
	**PC[a]**	**95%CI**	***p***	**PC[a]**	**95%CI**	***p***	**PC[a]**	**95%CI**	***p***	**PC[a]**	**95%CI**	***p***	**PC[a]**	**95%CI**	***p***
Sapporo	0.33	−1.40–2.08	0.712	2.12	0.32–3.96	0.021	2.17	0.43–3.93	0.014	1.05	−0.58–2.71	0.208	1.64	−0.42–3.75	0.120
Sendai	1.23	−0.79–3.30	0.234	0.43	−1.66–2.55	0.690	2.40	0.31–4.52	0.024	1.10	−0.87–3.10	0.276	0.97	−1.39–3.40	0.422
Saitama	−0.79	−2.71–1.17	0.429	−0.64	−2.61–1.36	0.525	0.30	−1.68–2.32	0.769	0.20	−1.73–2.18	0.839	−0.98	−3.28–1.38	0.412
Tokyo's 23 wards	−0.53	−1.24–0.18	0.144	−0.10	−0.82–0.63	0.793	0.51	−0.22–1.24	0.172	0.40	−0.31–1.12	0.271	−0.45	−1.31–0.40	0.298
Yokohama	0.69	−0.48–1.88	0.248	0.92	−0.27–2.13	0.131	1.65	0.45–2.87	0.007	0.46	−0.71–1.65	0.440	1.09	−0.30–2.50	0.125
Kawasaki	1.14	−0.42–2.71	0.152	0.80	−0.78–2.40	0.324	1.22	−0.36–2.84	0.131	0.82	−0.74–2.40	0.304	1.38	−0.48–3.28	0.147
Nagoya	0.15	−1.03–1.34	0.808	0.83	−0.36–2.03	0.173	1.07	−0.10–2.26	0.073	0.40	−0.76–1.57	0.503	0.67	−0.72–2.08	0.344
Kyoto	0.39	−1.06–1.87	0.596	0.88	−0.58–2.36	0.239	0.59	−0.85–2.06	0.424	0.10	−1.33–1.54	0.894	0.83	−0.84–2.52	0.331
Osaka	0.91	−0.04–1.87	0.060	−0.24	−1.19–0.72	0.626	0.27	−0.69–1.23	0.584	−0.13	−1.07–0.83	0.793	0.44	−0.65–1.54	0.434
Kobe	−0.46	−1.87–0.96	0.521	−1.29	−2.70–0.14	0.076	−0.60	−2.00–0.82	0.405	−1.24	−2.61–0.16	0.081	−1.17	−2.77–0.45	0.156
Hiroshima	−0.38	−2.07–1.34	0.661	0.29	−1.43–2.03	0.745	0.83	−0.88–2.57	0.342	−0.11	−1.79–1.61	0.903	−0.07	−1.99–1.88	0.941
Fukuoka	0.71	−0.59–2.02	0.287	1.67	0.36–2.98	0.012	0.28	−0.99–1.56	0.665	−0.29	−1.54–0.97	0.646	1.61	0.10–3.14	0.037

[a]Adjusted for calendar time, temperature, relative humidity, public holidays, and the day of the week. PC: percent change.

Suppl. Table S5. National average percent change in all-cause hospital admissions, total charges, and daily mean total charges per patient with a 10-μg/m^3 increase in ambient $PM_{2.5}$ concentrations adjusted using two-pollutant models (lag 01 for hospital admission and total charges, and lag 2 for the daily mean total charges per patient)

Variable	Hospital admissions			Total charges			Daily mean TCPP		
	PC[a]	95%CI	*p*	PC[a]	95%CI	*p*	PC[a]	95%CI	*p*
+Adjusted for coarse PM at lag 01	0.70	0.18–1.22	0.009	1.22	0.29–2.17	0.010	0.68	0.29–1.07	<.001
+Adjusted for O_x at lag 01	0.57	0.13–1.00	0.011	1.16	0.41–1.92	0.003	0.75	0.31–1.19	<.001
+Adjusted for NO_2 at lag 01	1.11	0.57–1.66	<.001	1.46	0.61–2.31	<.001	0.71	0.31–1.10	<.001
+Adjusted for SO_2 at lag 01	0.56	0.02–1.11	0.041	0.58	-0.41–1.57	0.255	0.70	0.27–1.12	0.001

[a]Adjusted for calendar time, temperature, relative humidity, public holidays, and the day of the week. TCPP: total charges per patient, PC: percent change, PM:particulate matter, O_x: photochemical oxidants, NO_2: nitrogen dioxide, SO_2: sulfur dioxide.

Suppl. Table S6. National average percent change in all-cause hospital admissions, total charges, and daily mean total charges per patient with a 10-µg/m³ increase in ambient $PM_{2.5}$ concentrations with alternative *df* values (lag 01 for hospital admission and total charges, and lag 2 for the daily mean total charges per patient)

Variable		Hospital admissions			Total charges			Daily mean TCPP		
	df	PC[a]	95%CI	*p*	PC[a]	95%CI	*p*	PC[a]	95%CI	*p*
Calendar time	5	0.64	0.23–1.05	0.002	1.00	0.26–1.74	0.008	0.50	0.12–0.89	0.009
	6	0.60	0.19–1.01	0.004	0.81	0.07–1.55	0.032	0.45	0.06–0.83	0.023
	8	0.55	0.14–0.97	0.009	1.11	0.37–1.86	0.003	0.68	0.26–1.09	0.001
	9	0.46	0.04–0.89	0.033	0.95	0.20–1.71	0.014	0.60	0.19–1.01	0.004
Temperature	2	0.61	0.18–1.05	0.006	1.32	0.60–2.06	<.001	0.85	0.43–1.28	<.001
	3	0.58	0.14–1.02	0.010	1.23	0.53–1.93	<.001	0.84	0.44–1.25	<.001
	4	0.57	0.16–0.99	0.006	1.14	0.42–1.87	0.002	0.69	0.29–1.08	<.001
Relative humidity	2	0.59	0.17–1.00	0.005	1.21	0.48–1.94	0.001	0.74	0.34–1.14	<.001
	4	0.55	0.13–0.97	0.011	1.19	0.45–1.94	0.002	0.77	0.37–1.17	<.001
	6	0.55	0.12–0.98	0.012	1.18	0.44–1.92	0.002	0.75	0.36–1.15	<.001

[a]Adjusted for calendar time, temperature, relative humidity, public holidays, and the day of the week. TCPP: total charges per patient, df: degree of freedom, PC: percent change.
